# Rheumatism

**Published:** 1855-01

**Authors:** 


RHEUMATISM.—When delirium occurs in acute rheumatism it is of grave import. Examine the heart carefully,—there is very likely to be pericarditis upon which the delirium depends. Bleed generally or locally according to circumstances, give calomel and opium, with salines and colchicum. Blister the cardic region well, both in front and back. At the same time support the patient with broth, and even wine if necessary; always remembering that many of these apparently inflammatory diseases are asthenic and not sthenic.—*Ibid.*

